# Enlarged uterine fibroid forming uterine diverticulum during pregnancy: a case report

**DOI:** 10.1186/s12884-020-03505-7

**Published:** 2021-01-07

**Authors:** Erina Akashi, Tatsuya Ishiguro, Taro Nonaka, Akiko Kobayashi, Koichi Takakuwa, Takayuki Enomoto

**Affiliations:** Department of Obstetrics and Gynecology, Niigata University Medical and Dental Hospital, 1-757, Asahimachi-dori, Chuo-ku, Niigata, 951-8510 Japan

**Keywords:** Uterine fibroid, Uterine diverticulum, Pregnancy

## Abstract

**Background:**

Although uterine fibroids are a common gynecologic neoplasm, uterine diverticulum accompanied by a uterine fibroid is unique. In addition, pregnancy complicated with uterine diverticulum is extremely rare. We experienced a case of a uterine fibroid that was associated with a uterine diverticulum that enlarged during pregnancy and puerperium.

**Case presentation:**

A 25-year-old nulligravida woman had an abnormal uterine cavity surrounded by myomatous mass. After natural conception, the mass and pouch had enlarged during pregnancy. Six months after elective cesarean delivery, she underwent laparotomy because of abdominal pain caused by the myomatous mass and the fluid inside. The tumor was connected to the midline of the posterior wall of the normal uterus. The resected tumor was pathologically diagnosed as leiomyoma and diverticulum.

**Conclusions:**

Pregnancy can stimulate uterine fibroids to form uterine diverticula. Resection of the diverticulum and fibroid is a useful option for symptomatic patients with desired future fertility.

## Background

Fibroid is the most common benign tumor that arises from the female genital tract, affecting 70–80% of women. Although uterine fibroids can cause several clinical symptoms, including severe menstrual bleeding, painful menstruation, bowel dysfunction, infertility, and miscarriage, most cases are asymptomatic [1]. Moreover, various sized fibroids can distort and elongate the shape of the uterus. Fibroids are considered estrogen/progesterone-dependent and pregnancy may influence their size, which tends to enlarge, especially during the first trimester [2]. We experienced a unique case of a uterine fibroid that was associated with a uterine diverticulum that enlarged during pregnancy and puerperium.

## Case presentation

**Fig. 1:**
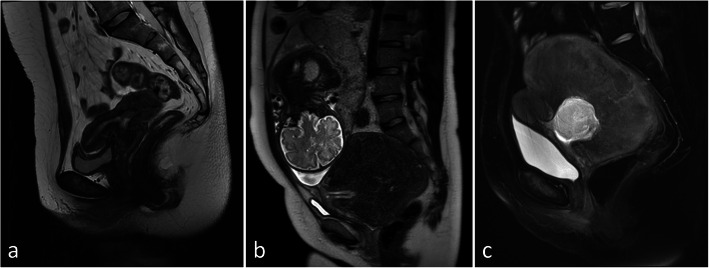
T2-weighted magnetic resonance imaging (MRI) of the uterus. MRI shows a myomatous mass with homogenous intensity from the normal uterine posterior wall close to the internal ostium of the uterus, which shaped the inner pouch structure (**a**). The myomatous mass increases at gestational week 32 (**b**). Post-cesarean section, the myomatous mass is as large as that observed during pregnancy, with an increase in the inner fluid (**c**)

A 25-year-old Japanese nulligravida woman was referred to our hospital to evaluate the abnormal structure of her uterus from a nearby clinic. She had no history of gynecological diseases, procedures, or uterine trauma. Her menstrual cycle was regular without any symptoms. Magnetic resonance imaging (MRI) revealed a myomatous mass with homogenous intensity present on the posterior uterine wall close to the internal ostium of the uterus, which shaped the inner pouch structure that was filled with a small amount of fluid (Fig. 1a). Upon initial examination, it appeared to be a rudimentary uterine horn; however, hysterosalpingography showed the patency of bilateral fallopian tubes connected with the fundus of the anterior uterus.

Although the pregnancy was uneventful after natural conception, the myomatous mass and the pouch had become enlarged (Fig. 1b). At 38 + 4 gestational weeks, the patient underwent an elective cesarean delivery because the enlarged mass interfered with a vaginal delivery. Although involution of the uterus was uneventful and her menstruation did not occur during the four months after delivery, she presented at our department again with lower abdominal pain. The normal uterus was empty and about 7 cm in size. MRI showed that the myomatous mass was just as large as it was during pregnancy, and the fluid inside the myomatous mass had increased (Fig. 1c).

**Fig. 2:**
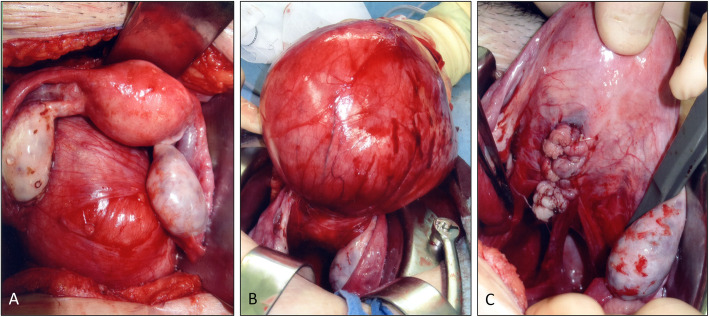
Intraperitoneal findings during surgery. The tumor is located behind the uterus, covered with thin serosa (**a**), and connected with the midline of the uterus (before (**b**) and after (**c**) resection of the tumor)

**Fig. 3:**
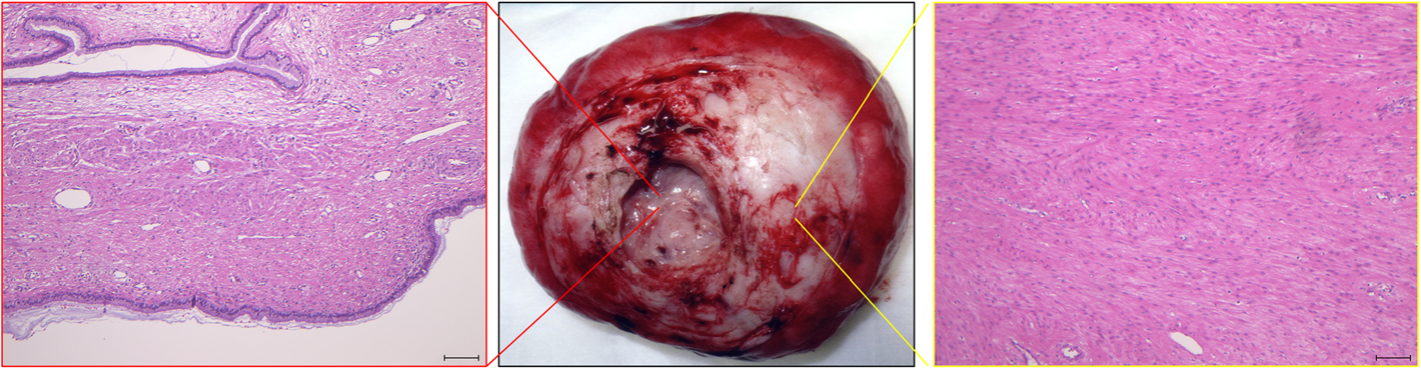
Histological findings of the resected diverticulum and tumor. Hematoxylin–eosin staining images detect leiomyoma (right). The inner pouch space is covered with inner cervical mucosa accompanied with inner cervical glands (left)

To improve her abdominal symptoms, she underwent laparotomy six months after delivery. During laparotomy, the uterus was completely normal with normal fallopian tubes and ovaries. A hard mass was found behind the uterus covered by thin serosa (Fig. 2a) and connected to the posterior uterine wall in the mid-line (Fig. 2b). The root of the tumor was about 3 cm in diameter. The tumor was taken out after incision of the root. The resected mass was about 18 cm in diameter and weighed approximately 1100 g. Macroscopically, the mass was a homogenous white pink hard tumor. The microscopic histological diagnosis was leiomyoma. The inner pouch space was covered with inner cervical mucosa, with inner cervical glands filled with a brownish mucosal fluid (Fig. 3). The final diagnosis was uterine fibroid and diverticulum. After surgery, her abdominal symptoms resolved, and her regular menstruation resumed for six months.

## Discussion and conclusions

This report presented a unique uterine diverticulum case with enlarged uterine fibroid during pregnancy and puerperium. A uterine diverticulum is a rare occurrence and is divided into congenital and secondary diverticula. Secondary uterine diverticula can occur after uterine instrumentation or trauma, including cesarean section, which can cause iatrogenic diverticula to occur on the weak site of the uterus. On the other hand, true congenital uterine diverticula, which occurs at a weak site of the uterine wall during development [3], is an extremely rare anomaly [4]. 21 cases of congenital uterine diverticulum were previously reported [3–13]. Our case was suspected to be a congenital uterine cavity worsened by a uterine fibroid and pregnancy because of several reasons. (1) The presence of the diverticulum in the midline around the uterine isthmus which is the weak site of the two fused Mullerian ducts [14]. Most of the previous reports confirmed the presence of the congenital diverticulum in the midline around the uterine isthmus [3–5]. (2) The studied woman with the nulli-para had no history of gynecological procedures or uterine trauma, which is often the cause of secondary diverticulum. (3) Usually, the congenital diverticulum pouch is covered by epithelial cells connected to the uterine cavity (the surgically excised diverticulum of the studied case was connected to the uterine cavity and was covered by cervical mucosa) [3].

Several factors stimulate the weak myometrium site of the uterus to form a diverticulum. Some reports showed that dilation of the cavity can occur during pregnancy and labor [4]. A similar case of uterine diverticulum with diffuse fibroid in a nulligravida women was reported recently [5]. In the studied case, we supposed that pregnancy and uterine fibroid cooperatively stimulated uterine wall and facilitated the enlargement of the congenital uterine diverticulum. The uterine muscle tried to expel the large fibroid from its anatomical locus even when not pregnant. Especially during pregnancy, the muscle contractions necessary for uterine growth promoted the function. As a result, the fibroid was dragged out of the uterine muscular axis, which led to the formation of a muscular hernia dragged into part of the endometrial mucosa of the uterus. Like these conditions, the results of ruptured degenerated fibroid or surface vessel cause hematoperitoneum even during pregnancy[15–17].

Uterine diverticulum causes infertility. Seound et al. described the mechanisms of diverticulum-associated infertility as storage of motile spermatozoa at the diverticulum and the adverse function of old blood in the diverticulum to spermatozoa and normal endocervical gland [6]. In this case, fortunately, the patient conceived naturally despite these obstacles. In addition, the diverticulum also causes pregnancy complications. It was reported that a uterine diverticulum can cause the rupturing of a uterus [18, 19] and might play a partial role in the premature rupturing of the membrane [4]. In the studied case, there was no perinatal complication related to the diverticulum. This explained by the presence of the large fibroid, which prevents entrapment of fetal parts, membranes in the diverticulum and prevents uterine rupture. The delivery mood is another controversial topic in uterine diverticulum cases, because of the diverticulum size and position. Nonetheless, cesarean section should be performed in cases of large uterine diverticulum regardless of the presence of an associated uterine fibroid.

Because of the rare nature of uterine diverticulum, there is no consensus treatment. Hysterectomy is a treatment option for symptomatic patients with no desired future fertility; otherwise, diverticulectomy is a useful treatment option for patients with few symptoms who desire future fertility. Women managed by divertculectomy should be counseled about possible risks of uterine rupture in future pregnancy. Surgical resection is one of the treatment options for the management of a large diverticulum.

In conclusion, we present a rare case of uterine diverticulum complicated with a uterine fibroid during pregnancy. Uterine diverticulum should be considered in the differential diagnosis of abnormal uterine cavity, and resection of diverticulum is a useful treatment option for women who desire future fertility.

## Data Availability

All data analyzed during this study are included in this report.

## Abbreviation

MRI: Magnetic resonance imaging
